# In multiple myeloma, bone-marrow lymphocytes harboring the same chromosomal abnormalities as autologous plasma cells predict poor survival

**DOI:** 10.1002/ajh.23194

**Published:** 2012-03-12

**Authors:** Carina S Debes Marun, Andrew R Belch, Linda M Pilarski

**Affiliations:** Department of Oncology, University of AlbertaEdmonton, Alberta, Canada

## Abstract

Chromosomal abnormalities in plasma cells (PCs) from multiple myeloma (MM) provide a clonal signature to identify malignant cells. BM-lymphocytes from MM aspirates, defined by stringent criteria, were screened for the same chromosomal abnormalities as autologous PCs, including translocations, deletions, and amplifications. For 200 MM patients, we evaluated BM mononuclear cells to identify lymphocytes and autologous PCs on the same slide, followed by interphase fluorescence in situ hybridization to characterize their chromosomal abnormalities. Of all patients having a given chromosomal abnormality(s) in PCs, 45% showed that same abnormality(s) in 2–37% (median = 5%) of BM-lymphocytes. Most translocations, amplifications, and deletions found in MM PCs were also detected in lymphocytes, above the healthy-donor “cut-off.” In patients having chromosomally abnormal CD20^−^ PCs, chromosomally abnormal lymphocytes were found among CD20+ cells confirming them as B cells. Exceptions were amplification of 1q21 or p53 deletion, which characterize PCs but were undetectable in BM-lymphocytes, suggesting that processes leading to these abnormalities may be exclusive to PCs. For a set of 75 patients whose BM-lymphocytes and PCs were analyzed by all six probe sets, 58% of those with abnormal PC also had abnormal BM-lymphocytes harboring from one to five different abnormalities. Confirming the clinical significance of chromosomally abnormal BM-lymphocytes, MM patients having abnormalities in both lymphocytes and PC had significantly worse survival than those with abnormalities only in PC (HR = 2.68). The presence of at least one chromosomal abnormality in BM-lymphocytes appears to have greater clinical significance than particular abnormalities. Chromosomally abnormal BM-lymphocytes correlate with poor outcome and by extrapolation with more aggressive disease.

## Introduction

Multiple genetic abnormalities have been described in multiple myeloma (MM), including numerical abnormalities and structural changes such as translocations, duplications, inversions, and amplifications. The most common numerical change is monosomy of chromosome 13 [1–5] with infrequent interstitial deletions [6, 7] and trisomies of chromosomes 3, 5, 9, 11, 15, and 21 [5,8]. The most common structural abnormalities are translocations of IgH locus and amplification of 1q21 [9]. The IgH translocation partner remains unidentified in roughly 50% of MM cases having a 14q32 translocation. Several recurrent IgH translocation partners have been identified, including 11q13 (15–20%), 4p16 (5–15%), 16q23 (2–5%), 6p21 (5%), and 20q12 (2%) [10–16]. Some chromosomal abnormalities appear early in malignant development, such as the primary IgH translocations or deletion of chromosome 13, and others appear to be late acquisitions as the MM clone evolves, such as p53 deletion or 1q21 amplification [1, 17]. Although no unique abnormality characterizes all MM, those harbored by the plasma cell (PC) in each patient represent clonotypic markers to identify clonal relationships within that MM patient.

Although PCs mediate the pathology of MM, a substantial body of evidence indicates that the malignant clone also includes B lymphocytes that share the clonotypic IgH VDJ gene rearrangement characterizing autologous PC [18–25]. The MM clone includes not only PCs but also late-stage B cells [21] and preswitch B cells [22–25]. MM B lymphocytes are DNA aneuploid [19], have autocrine cytokine networks [26], and may mediate malignant spread [27]. Clonogenic population that are phenotypically and physically distinct from PCs include presumptive MM cancer stem cells (CSCs) [28, 29]. CD20+ clonotypic lymphocytes from 3D culture of ex vivo MM BM are self-renewing and after serial passages give rise to PCs [30], as expected for CSC. Furthermore, population that include B lymphocytes but not PC xenograft human MM to immunodeficient mice [22, 31, 32], as do progenitor population that lack B-cell markers [33]. This suggests that MM CSC may include multiple compartments of clonotypic cells at presumptively sequential stages of differentiation with different generative capability [34].

Chromosomal abnormalities in PCs provide one or more clonotypic marker(s) distinct from the IgH VDJ rearrangement. Most studies analyze only purified PCs, excluding all other components of the MM clone. Using stringent criteria, we here analyzed BM-lymphocytes from 200 MM patients for chromosomal abnormalities that characterize autologous PCs. This strategy ensures that the chromosomal abnormalities in lymphocytes and PCs are evaluated on the same slide, with identical staining conditions, with adjacent nonmalignant cells serving as internal controls. About 15% of MM patients have CD20+ PC. After selecting patients whose clonal PCs were reported CD20−, we found that at least some of the chromosomally abnormal lymphocytes were CD20+ B cells. However, the MM clone is heterogeneous, and MM CSC capability may be multifactorial [34]. Analysis of lymphocytes instead of purified B-cells enabled inclusion of most B-cell subsets, while avoiding assumptions about the nature of MM CSC. Purification of B-cells would inappropriately exclude cells that have lymphocyte morphology but lack B-cell markers as well as any dedifferentiated PC having lymphocyte morphology. The validity of this strategy is verified by our observation that chromosomally abnormal BM-lymphocytes correlate with significantly reduced survival, independent of treatment(s) or type of abnormality.

## Materials and Methods

### 

#### Patients

BM samples were collected from 200 randomly selected MM patients (Table I) in accord with the Declaration of Helsinki, with the approval from Institutional Review Boards of the University of Alberta and Alberta Health Services and written informed consent. For 175 patients, the samples analyzed by fluorescence in situ hybridization (FISH) were pretreatment diagnostic BM. For 25 patients, BM aspirates were taken during or after treatment; 11 of these patients were in relapse and 14 were on treatment, including VAD, lenolidomide, bortezomib, and dexamethasone in different treatment protocols.

**TABLE I:** Clinical Parameters of MM Patients

Patients	200
Age median (range)	64 (33–90)
Sex (male %)	61.5
Isotype (%)	
IgG	61.8
IgA	21.5
LC	10.2
Other/unknown	6.5
light chain (K %)	60
% BM plasmocytosis median (range)	40 (2–100)
g/L M protein median (range)	22.5 (0–106)
mg/L β2 microglobulin median (range)	4.2(1.44–113)
g/D Urine LC median (range)	0.39 (0–5.19)
g/L Albumine median (range)	43 (21–77)
U/L LDH median (range)	441 (213–1908)
g/L Hemoglobin median (range)	113 (72–171)
μmol/L Creatinine median(range)	99 (45–959)
mmol/L Calcium median (range)	2.36 (1.63–3.83)

IgG, immunoglobulin G; IgA, immunoglobin A; LC, light chain; BM, bone marrow; M, monoclonal; LDH, lactate dehydrogenase.

#### Cytomorphology and FISH

Cytospin slides were prepared from the BM mononuclear cells obtained by density gradient centrifugation (Ficoll–Hypaque) as previously described [19], followed by May Grünwald–Giemsa staining (MGG) [35, 36]. Using the Bioview Duet (Revohot, Israel), automated scanning of each slide identified all lymphocytes and PCs, based solely on morphology, and their positions on the slide, verified by visual inspection, were recorded. Monocytes (M) and/or polymorphonuclear (PMN) cells on the same slide served as internal negative controls. Lymphocytes were evaluated on the same slide as the positive (PC) and negative control (e.g., PMN) population, thereby ensuring identical staining conditions for test and control-cell types. MGG was then destained using ice-cold methanol:acetic acid (3:1) as previously described [35, 36].

Stringent criteria were used to define lymphocytes: cell size between 7 and 12 μm, high nucleus to cytoplasm ratio (N/C ratio), nucleus oval or slightly indented, dense clumped homogenous chromatin devoid of nucleolus, little rough endoplasmic reticulum (RER) light blue basophilic cytoplasm from scanty to medium, mostly agranular, but including large granular lymphocytes. These criteria exclude PCs, plasmablasts, and lymphoplasmocytic cells by their smaller N/C ratio, much denser and patchy chromatin condensation, increased cytoplasm and cytoplasmic basophilia, and increased amounts of RER that define the characteristic perinuclear zone. Monocytes are excluded by their smaller N/C ratio, nonhomogeneous chromatin, larger size, indented nucleolus, and gray, less basophilic cytoplasm with dusty granulation. Blasts were excluded by their higher N/C ratio, immature open chromatin, higher cytoplasmic basophilia and presence of nucleolus, and sometimes larger size. The criteria also exclude all granulocyte and red-cell precursors. None of the patients with abnormalities in their lymphocytes had “small lymphocyte-like” PC myeloma [37], and only two were reported to have lymphoplasmocytic morphology.

Fluorescence in situ hybridization (FISH) was performed to detect the most common genetic abnormalities present in MM patients. Probes were from Vysis (Abbot Molecular) through Intermedico (Ontario, Canada) unless otherwise noted. D13S319 was used to detect the deletion of chromosome 13 (13 probe), a mixture of LSI p53 and D17Z1 (CEP17, centromeric probe) to detect p53 deletion (p53 probe), LSI IgH dual color break apart probe to detect presence of any translocation of the IgH locus (BA probe), LSI IgH/CCND1-XT DF to detect translocation t(11;14)(q13;q32) (11;14 probe), and LSI IgH/FGFR3 DF to detect translocation t(4;14)(p16;q32)(4;14 probe). To detect amplification of the 1q21 locus that includes the CKS-1B gene, probes were provided by Dr. John Shaughnessy [17] to test the first 20 patients. We then developed our own probe of DNA from BAC-clone RPCI-11-307C12 labeled with a Vysis kit, coupled with a commercial α-satellite probe targeting centromere of chromosome 1 (D1Z5) (ch1 probe); the remaining patients were assessed using this latter set of probes. Both probes gave similar results. FISH was performed as previously reported with minor changes [36]. Published protocols were used for the 1q21 probe provided by Dr. Shaughnessy [17].

Overall, for the first 100 patients, PC and lymphocytes were assessed on the same slide regardless of the presence or absence of abnormalities. For the following 100 patients, the lymphocytes were assessed only if autologous PCs on the same slide were found to have chromosomal abnormalities.

To categorically identify B lymphocytes but avoid morphological changes that accompany staining and sorting, for seven randomly selected patients, cytospin slides were immunostained with anti-CD20 to identify the lymphocytes harboring chromosomal abnormalities using clone L26 (mouse antihuman CD20) from Dako (Denmark), HRPO polyclonal against mouse antibody (Dako envision system), and AEC (3-amino-9-ethylcarbazol) for color development (Dako) as per manufacturer's instructions. We recorded the location of the CD20+ cells and performed FISH as previously described. We also performed FICTION (fluorescent immunophenotyping and in situ cytogenetics) on five selected patients (different from the immunostained ones) using L26, detected with Marina Blue antimouse (Molecular Probes-Invitrogen Canada, Burlington, ON), fixation in 2% paraformaldehyde for 5 min at RT and FISH as described earlier. These methods are complex but confirm that at least a proportion of abnormal lymphocytes are B cells.

#### Control studies

Based on FISH performed on lymphocytes in healthy individuals, an independent cut-off percentage (median plus three standard deviations) was calculated for each probe set. This defined “background” staining for lymphocytes in peripheral blood from 5 to 10 healthy donors, using the same protocol described for the patient samples. Briefly, the cytospin slides were stained with MGG, lymphocyte locations were recorded, slides were destained, and FISH with the appropriate probe was performed. Subsequently 100–300 lymphocytes were analyzed on each slide to search for presumptive “false-positive” cells. The cut-off percentages for each probe were established to be 4% for the deletion of p53, 8% for the amplification of 1q21, 5% deletion 13, and 12% for the IgH break apart probe. For the dual-fusion probes, 1% was set as an arbitrary cut off, because no false positives were detected for either probe set in healthy lymphocytes.

As previously reported, about 40% of the patients with MM have hyperdiploidy and 22% hypodiploidy [5, 8]. When using any of the probes described earlier, amplifications or deletions of the specific chromosomal region are also detected. Although these abnormalities are not the ones that the probe was intended to detect, they are still clonal markers for the MM PC; therefore, lymphocytes showing the same pattern of abnormality as autologous PCs were also considered to be clonal. To decide if an alternative locus abnormality was significant (e.g., amplification of chromosome 4 detected with the 4;14 probe), we averaged the false positives detected in the lymphocytes of healthy donors and established a common cut off. Any chromosomal deletion was significant if detected in more than 4% of the lymphocytes, and any chromosomal amplification was significant if detected in more than 3% of lymphocytes. For the PC analysis, we followed the European Myeloma Network guidelines [38].

For all FISH-stained slides, normal hematopoietic cells (e.g., M and PMN) in the BM were screened as internal controls for the abnormalities detected by a given probe set. These were always below the designated cut off.

#### Statistical analysis

We assessed the clinical parameters with relation to the presence or absence of each type of abnormality in the BM lymphocytes. Chi-square test/Fisher's exact test was used to examine the individual effect of an abnormality in lymphocytes on each dichotomized clinical parameter. For the combined effect of multiple abnormalities in lymphocytes on each clinical parameter, a multivariate logistic regression model was used by first including all the abnormalities and keeping only those significant abnormalities in the final model. An odds ratio (OR) larger than one indicates that the chromosomal abnormality increases the chance of an abnormal clinical parameter. If OR is less than one, then the chromosomal abnormality decreases the chance of having an abnormal clinical parameter.

To determine whether or not a lymphocyte or PC abnormality was correlated with the time to death after diagnosis, we used the log-rank test. Survival curves were generated using the Kaplan–Meier method. The mean follow up for patients was 986 days (10–3801 days, median 896 days). Cox's proportional hazard model was used for multivariate analysis of abnormal lymphocytes as correlated with length of survival.

## Results

### Experimental approach

**Figure 1:**
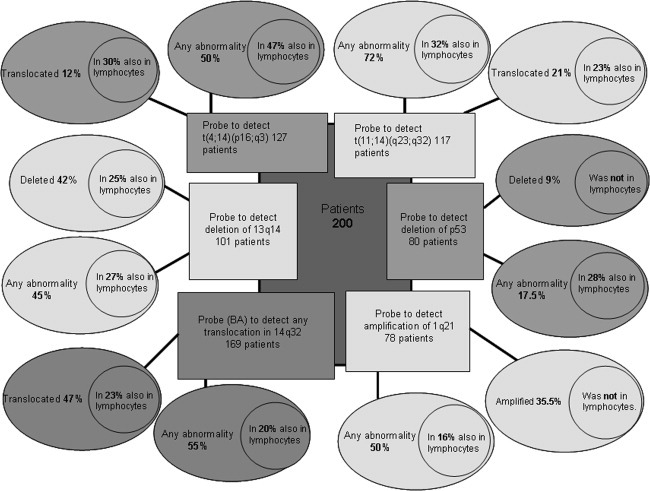
Genetic abnormalities detected in PC and BM-lymphocytes of patients with MM. Each probe is characterized by two large ovals. One corresponds to the abnormality that the probe was created to detect [such as t(4:14)] with the percentage of patients presenting this aberration. The second oval, termed “any abnormalities,” represents those abnormalities that the probe intended to detect plus any deletions or amplifications of the loci against which the probe is directed. For example: in the case of the probe for t(11;14), “any abnormality” conveys the following: translocation 11;14, amplification of 11q13, deletion of 11q13, amplification of 14q32, and deletion of 14q32 as detected by this probe. Each oval has a circle inside that shows the percentage of patients that share the same abnormality in both PC and lymphocytes.

We analyzed PCs and lymphocytes in BM from 200 consecutive MM patients: 169 for the BA probe, 101 forchromosome 13, 127 for t(4;14)(p16;q32), and 117 for t(11;14)(q13;q32). BM was analyzed from 80 patients for chromosome 17 (p53) and from 78 patients for chromosome 1, as summarized in Table II/Fig. 1. Lymphocytes were identified by stringent morphologic criteria that would include, for example, T, NK, B cells, lymphocytic MM cells lacking B-cell markers, and any “dedifferentiated” PC with lymphocyte-like morphology. MM-related lymphocytes harboring chromosomal abnormalities are expected to comprise only a portion of total BM-lymphocytes. For a subset of patients, immunostaining or immunofluorescence with CD20 preceded FISH: in most cases, abnormal cells expressing CD20 were seen, but non-CD20 cells with abnormalities were also detected. Analyzing lymphocytes, PCs and negative control cells on the same slide, in identical staining conditions, provides rigorous internal controls but likely *under*estimates the true frequency of chromosomally abnormal lymphocytes.

**TABLE II:** Abnormalities in BM-PC and BM-Lymphocytes

Probe	Detected pattern using the probe	% of patients with abnormality in PC	% of (+) patients whose BM lymphocytes have the same abnormality as PC	% of lymphocytes with abnormality. Median (range)
Detected by translocation probes				
**BA**	any IgH translocation	47%	23%	16% (13-20%)
	amplification 14q32	14%	9%	4%
	deletion 14q32	10%	18%	9% (5-37%)
	any abnormality	55%	20%	
**(4;14)**	t(4;14) translocation	12%	30%	3% (2-6%)
	amplification 4p16	5.5%	57%	6% (4-11%)
	amplification 14q32	20.5%	50%	9 % (4-18%)
	deletion 14q32	12%	20%	12 % (5-19%)
	amplification 4p16 plus amplification 14q32	5.5%	71%	8 % (4-13%)
	any abnormality	50%	47%	
**(11;14)**	t(11;14) translocation	21%	23%	5% (2-20%)
	amplification 11q23	26%	19%	8 % (4-15%)
	amplification14q32	12%	50%	7% (4-17%)
	deletion 14q32	5%	33%	16 % (5-27%)
	amplification 11q23 plus amplification14q32	12%	25%	7.5% (4-15%)
	any abnormality	72%	32%	
Detected by deletion probes				
**13q14**	deletion 13	42.5%	25.5%	7.7 % (6-30%)
	trisomy	3%	66%	12.5%(8-17%)
**p53**	deletion p53	9%	**0%**	
	trisomy 17	11%	33%	4% (4-5%)
**1q21**	amplification locus	35%	**0%**	
	trisomy	20.50%	31%	6% (4-34%)
	monosomy	2.5%	0%	

BM slides from 200 MM patients were separately stained with one or more different probes or probe sets; most were stained only with some of the indicated probes. BM slides from a subset of 75 patients were separately stained with all probe sets (see Results and Figure 4). One hundred and thirty-five patients had at least one detectable abnormality with the probe sets used. Amplification of the locus = more than two copies; deletion of the locus = less than two copies; any abnormality = all the abnormalities that could be detected by the probe including translocations, amplifications, and deletions. For six patients with amplification of at least three of the tested chromosomes (1, 4, 11, 14, and 17), although in PC, we detected two signals for chromosome 13, we still considered them as having a deletion following the European Myeloma Network guidelines [39]. Interestingly, three of these six patients have 6–8% of their BM-lymphocytes harboring three signals for ch13, suggesting that loss of the third ch13 occurred only in the PC population.

As determined by a centromeric-specific probe (see Methods section).

For BM PCs, the incidence of abnormalities was as expected. In PCs, we detected deletion 13 in 42.5% of the patients, 1q21 amplification in 35%, p53 deletion in 9%, t(4;14)(p16;q32) in 12%, t(11;14)(q13;q32) in 21%, and any translocation of 14q32 in 47% of the patients. In a set of 75 patients from whom we had sufficient BM to assess staining with all probe sets in parallel, 93% had PCs with at least one abnormality and 58% of those patients had at least one abnormality shared between PCs and lymphocytes.

Results from a given slide were included in the analysis only if identified abnormalities were below the designated cut off for autologous BM M/PMN cells on the same slide (internal negative control). Overall, BM M/PMN was always below cut off for abnormalities.

### Translocation probes

**Figure 2:**
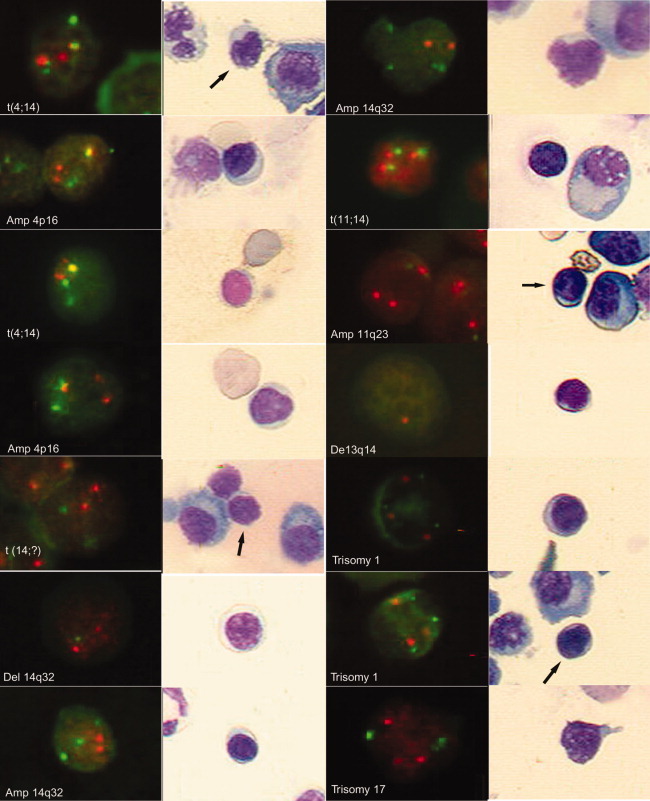
BM-lymphocytes with genetic abnormalities are detected by interphase FISH. All of the pictures were taken with the Bioview Duet Scanning System. In a set of two pictures, the one on the right is the MGG image of the picture on the left. The MGG images were taken using a Zeiss plan neofluar 40×/0.75 objective, and the fluorescent images were collected through a Zeiss plan-neofluar 63×/0.95 Korr dry lens. All the fluorescent images were adjusted in contrast and brightness, and in some of them, the gamma was also adjusted using Photoshop 6 software. The colors were not altered in any way. The cloning tool was used to erase the location mark that the software engraves in the picture in both the bright field and fluorescent images. The arrows point to the lymphocyte shown in the fluorescent picture. Left panel: from top to bottom. Red (R), Green (G), Fusion (F) = R, and G overlapping or very close to each other. For most of the probes, the normal pattern is 2R2G, except for BA (normal pattern 2F) and 13 probe (normal pattern 2R). (1) and (3) Translocation t(4;14) (2F1R1G) detected by 4;14 probe (ch 4 in R and ch 14 in G). (2) and (4) Amplification 4p16 (1F2R1G) detected by probe 4;14. (5) Translocation 14q32 (2R2G) detected by BA probe (ch.14q32 labeled R and G, when separated indicates translocation). (6) Deletion of part of 14q32 locus (2R1G) detected by a BA probe described earlier. (7) Amplification 14q32 (2R3G) detected by 11;14 probe (ch 11 in R and ch 14 in G). Right panel, from top to bottom. Amplification 14q32 (2R4G) detected by 11;14 probe described earlier. Translocation t(11;14) (3F) [the normal translocation pattern would be 2F1R1G, but in this case, the nontranslocated chromosomes (R and G) are likely overlapped simulating another F] detected by 11;14 probe. Amplification 11q23 (3R2G) detected by 11;14 probe. Deletion 13q14 (1R) detected by 13 probe (13q14 locus in R). (5) and (6) Trisomy 1 (3R3G) detected by the ch1 probe (CEP 1 in R and 1q21 in G). (7) Trisomy 17 (3R3G) detected by p53 probe (CEP 17 in G and p53locus in R).

**Figure 3:**
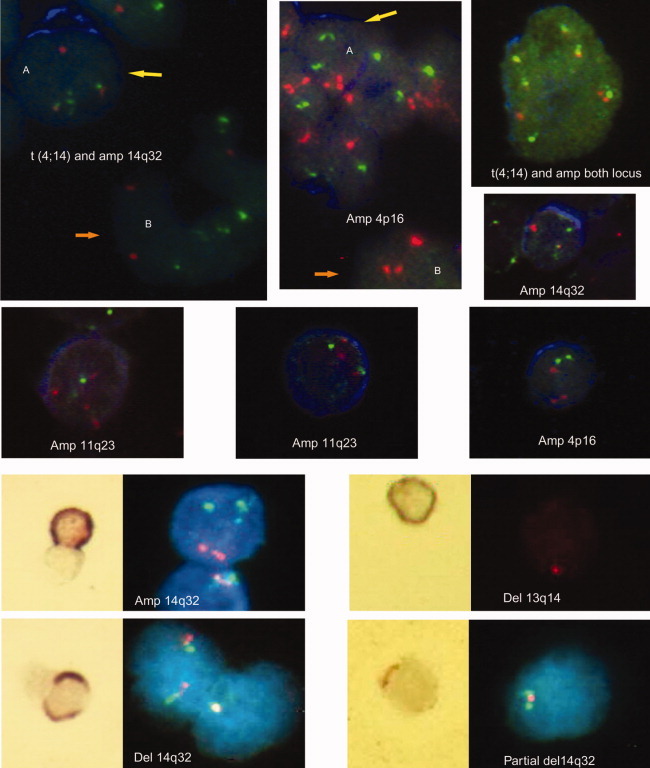
CD20-positive B cells with genetic abnormalities. All the pictures were taken with the Bioview Duet Scanning System. The IHC images were taken using a Zeiss plan neofluar 40×/0.75 objective, and the fluorescent images were collected through a Zeiss plan-neofluar 63×/0.95 Korr dry lens. CD20 immunostaining was done on 7 MM patients by IHC and five different patients by FICTION. All these patients had BM plasma cells *lacking* expression of CD20, ensuring that CD20 identified only B cells. The panels in the top two rows show FICTION staining; CD20-positive cells have blue cytoplasm. All the images show abnormal FISH patterns in CD20+ cells, as indicated on each panel. In the first and second images (top row, left to right), the yellow arrow points a genetically abnormal CD20+ cell (A), and the orange arrow points to a genetically abnormal CD20 negative cell (B). All patients had CD20-negative PC as shown by immunostaining of the BM biopsies. The panels in the bottom two rows show CD20+ IHC followed by FISH. The IHC image is on the left (brown stain for CD20), and the fluorescent FISH image with blue DAPI staining to indicate the nucleus is on the right. Red (R), Green (G), Fusion (F) = R, and G overlapping or very close to each other. For most of the probes, the normal pattern is 2R2G, except for BA (normal pattern 2F) and 13 probe (normal pattern 2R). FICTION PANEL (rows 1 and 2): by column, from top to bottom. A: Translocation t(4;14) and amplification 14q32(2F1R3G) detected by 4;14 probe. B: Amplification14q32 (2R4G) detected by 4;14 probe. Amplification 11q23 (4R2G) detected by 11;14 probe. A and B: Amplification 4p16 (3R2G) detected by 4;14 probe. Amplification 11q23 (3R2G) detected by 11;14 probe. Translocation t(4;14) and amplification of both loci (4F2R2G) detected by 4;14 probe. Amplification 14q32 (1F1R2G) detected by 4;14 probe. Amplification 4p16 (3R2G) detected by 4;14 probe. IHC panel (rows 3 and 4): left side, top to bottom. Amplification 14q32 (2R3G) detected by 11;14 probe. Deletion 14q32 (1F) detected by BA probe. Right side, top to bottom. Deletion 13q14 (1R) detected by 13 probe. Partial deletion of 14q32 (1F1G) detected by BA probe.

To assess for translocations of 14q32, we used a BA probe (“break apart”) that detects any translocations on 14q32, a dual-fusion t(4;14) probe, and a dual-fusion t(11;14) probe. The dual-fusion probes detect two different chromosomal regions, one in 14 and one in 4 or 11, respectively, so that amplifications (amp.) or deletions (del.) in either locus were recorded. For example, a patient having trisomy 11 will show amplification for the 11q13 probe, which we designate as amp11. The abnormalities detected in BM-lymphocytes are summarized in Table II. Figure 2 demonstrates lymphocytes with genetic abnormalities. Figure 3 shows that for a subset of patients whose BM cells were stained with CD20, some abnormal lymphocytes were CD20+.

For unbalanced translocations (25% of the translocations detected by the 4;14 or 11;14 probe sets), we were usually unable to report lymphocytes with the same translocation. This is because an observed pattern of 1F1G1R is frequently regarded as overlapping signals unless the percentage of cells presenting this pattern is above 20%. Nevertheless, we were able to detect this pattern in 3 of 14 patients, affecting 19–29% of BM-lymphocytes.

### BA probe

The BA probe was used for BM from 169 patients, identifying an IgH translocation in 79 patients (47%), in 14–100% of PCs (median = 78%). For 18 (23%) of those patients presenting the abnormality in PCs, we found the same abnormality in the BM lymphocytes. One 14q32 was deleted in the PCs of 17 (10%) of the patients, with the same abnormality in the lymphocytes from three (18%) of them. Likewise, amplification of the 14q32 was detected in 23 (14%) patients with BM-lymphocytes bearing the same abnormality in two (9%) of them. In nine patients, this probe was not informative.

Taking into account all the abnormalities detectable with the BA probe, at least one was seen in PCs from 55% of the patients and the same abnormality in BM-lymphocytes from 20% of them.

### t(4;14)

BM from 127 patients was assessed. Overall, patients having t(4;14) comprised 25% of BA positive patients and 12% of the total cohort. Twenty patients had t(4;14) in 18–96% of PCs (median = 88%) with 6 (30%) of them also having the abnormality in 2–6% of the BM-lymphocytes (median = 3%).

Twenty-six patients (20.5%) showed amplification of ch14 and seven (5.5%) amplification of ch4 in PCs, with BM-lymphocytes also bearing the same abnormalities in 50 and 57%, respectively, of the same set of patients. There were 7 (5.5%) patients that had amplification of both loci in the same PCs, and 5 (71%) of them had dual amplification in the lymphocytes as well.

For deletions, one patient had only one copy of ch4 in PCs (also shown in the lymphocytes) but 15 (12%) with only one signal for ch14. Del14 was detected in the lymphocytes from three (23%) patients with del14 in BM-PC. Taking into account all abnormalities detected by this probe (translocations, amplifications, and deletions), we detected at least one abnormality in PC from 50% of the patients studied, with 47% of the same patients having identical abnormalities in their BM-lymphocytes (Fig 2). Some abnormal cells were CD20+ (Fig. 3).

### t(11;14)

Thirty-five patients (44% of BA positive patients and 21% of all patients studied) have detectable translocation (11;14) in their BM-PC, with 30–100% of positive PC (median = 79%). The same abnormality was observed in 2–7% of the BM-lymphocytes (median = 5%) in eight of those translocated patients (23%).

Amplification of 11q23 was detected in BM-PC from 31 patients (26%), 6 of whom also had amp11 in 4–15% of BM-lymphocytes. Amp14 was detected in 14 patients (12%) with 4–17% of the lymphocytes having the same aberration in seven of those patients (50%). We also found 14 patients (12%) with PCs having both amp11 and amp14; 25% of these patients had BM-lymphocytes harboring the same dual amplifications. We found del14 in six patients and of those, two (33%) show the same abnormality in their BM-lymphocytes.

Considering all of the abnormalities that can be detected with this probe, at least one abnormality was found in 72% of the patients tested, and for 32% of these patients, the same abnormality was found in the BM-lymphocytes (Fig. 2), including some that were CD20+ (from patients whose PCs were CD20−; Fig. 3).

### Deletion-amplification probes

We used three probes to detect deletions or amplification. To assess for the deletion of chromosome 13, we used D13S319 (regarded to be the most commonly deleted region in some studies [38]). A combination of a centromeric probe and a locus-specific probe was used to assess for p53 deletion and amplification of 1q21. Results are summarized in Table II.

### Deletion 13 (del13)

When del13 is detected, in most cases, this represents monosomy [15, 40]; in only a minority of cases does this represent an interstitial deletion. Hence we chose to utilize only one probe to assess for this abnormality. BM from 101 patients was analyzed. About 42.5% of patients showed del13 in 13–99% of BM-PC (median = 78%), and 11 (25.5%) of them also have the same abnormality in BM-lymphocytes (6–30%, median = 7%).

In three patients, we found amp13 in BM-PC (11–30% of PC). For two of these patients, amp13 was also detected in 8 and 17% of their BM-lymphocytes.

Overall, of 46% patients with ch13 abnormalities in PCs, 28% had the same abnormalities in their BM-lymphocytes (Fig. 2), including CD20+ cells (Fig. 3).

### Deletion of p53

We studied 80 patients and identified delp53 in seven patients (9%) in 43–98% of PC (median = 80%). We were unable to detect lymphocytes harboring delp53 for any of these patients. However, we did find nine patients (11%) showing trisomy 17 (detected by the centromere17 probe) and found the abnormality in 4–5% of the BM-lymphocytes in three of them (33%). This indicates that abnormal lymphocytes were present in the BM but did not acquire delp53, suggesting that delp53 may be generated by events that occur only in PC.

### Amplification of 1q21

Among 78 patients tested, amp1q21 was detected in 27 (35%) patients in 12–99% of PC (median= 77.5%). As was the case for delp53, we were unable to detect lymphocytes harboring amp1q21 above the cut-off threshold. In addition, two patients presented with monosomy 1 (detected by centromeric probe) in their PC, but the abnormality was not detected in their lymphocytes.

In contrast, 15 patients (19%) had PC with trisomy 1 and 1 patient had tetrasomy 1. For five of these patients (31%), we found the same abnormalities in their BM-lymphocytes. This indicates that BM-lymphocytes with ch1 abnormalities were present in BM of the affected patients, but they did not acquire amp1q21 or del1, suggesting that amp1q21 and del1 may be generated by events restricted to PC.

### Abnormal BM-lymphocytes are more frequent in patients whose PCs harbor multiple abnormalities

**Figure 4:**
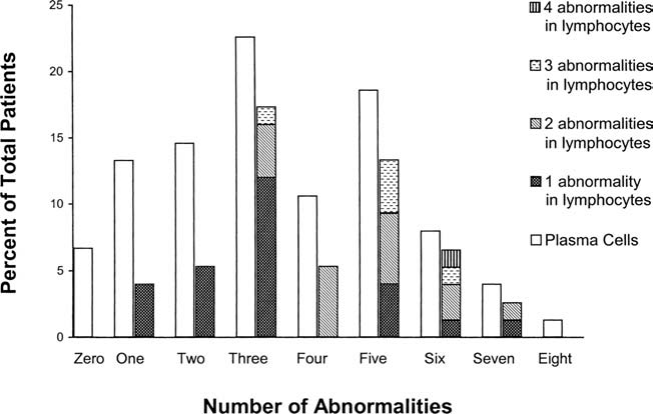
Detection of abnormal BM-lymphocytes increases in patients whose PC have multiple abnormalities. This chart was created using information from 75 patients for whom FISH was performed with all sets of the probes. The open bars represent the percentage of patients with zero, one, two, etc. abnormalities detected in the PC. The hatched bars correspond to the percentage of patients that bear the same abnormality(ies) in lymphocytes as those detected in autologous PC; hatched bars illustrate the percentage of patients with one, two, three, and four abnormalities in the lymphocytes as a cumulative height.

In a cohort of 75 patients, each tested for all probe sets, we determined the extent to which detection of abnormal BM-lymphocytes was dependent on the number of abnormalities found in autologous PCs (Fig. 4). For this cohort of 75, 93% had at least one abnormality in their PCs, as measured by the probe sets indicated earlier. Ten patients (13%) had only one abnormality in their PCs, and for these, BM-lymphocytes had the same abnormality in three of them (30%). Eleven patients (15%) had two abnormalities in their PCs, but the BM-lymphocytes from five of them had only one abnormality (45%). There were 17 (23%) patients with three abnormalities, and the same abnormalities were detected in BM-lymphocytes for 76% of them; 53% of these had one abnormality in the lymphocytes, 17% had two abnormalities in the lymphocytes, and 6% had three abnormalities in the lymphocytes. Eight patients (10%) had four abnormalities in their PCs; two abnormalities were detected in the BM-lymphocytes for 50% of them. There were 14 (19%) patients with five abnormalities in their PCs; the same abnormalities were detected in the BM-lymphocytes from 71% of them: 21% had one abnormality in BM-lymphocytes, 29% had two abnormalities, and 21% had three abnormalities. For those patients presenting with six abnormalities in their PCs (7%), we found one abnormality in BM-lymphocytes in 17%, two abnormalities in 33%, three abnormalities in 17%, and four abnormalities in 17%, totaling 83% of these patients having abnormal BM lymphocytes. There were only three patients, in whom we detected seven abnormalities in the PC, and ofthose one patient had one abnormality in their lymphocytes and a second had two abnormalities in their lymphocytes.

Overall, based on analysis of all six probe sets, 70 of 75 patients (93%) had at least one abnormality in their BM-PC and of these 58% had at least one abnormality present in their BM-lymphocytes.

### The presence of chromosomal abnormalities in BM-lymphocytes correlates with significantly reduced overall survival

**Figure 5:**
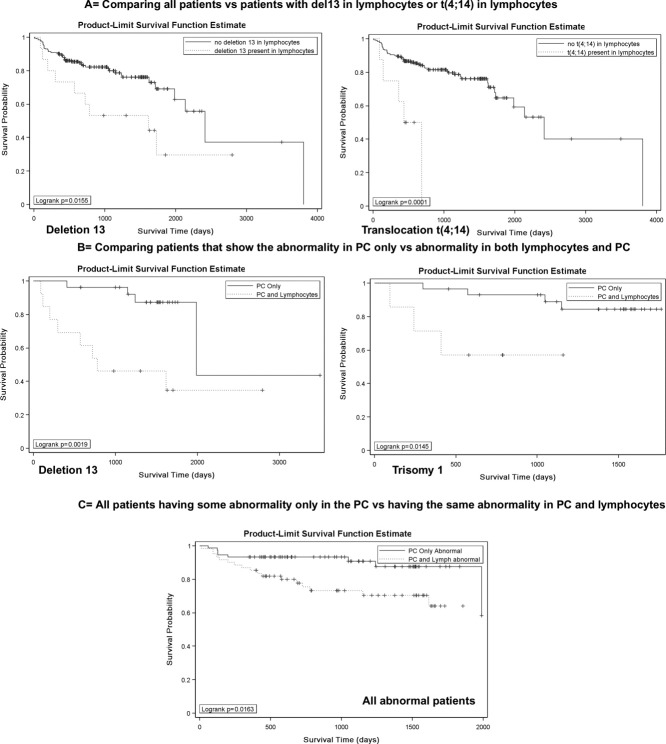
The presence of chromosomally abnormal BM-lymphocytes correlates with reduced survival. Panel A: Aggregate overall survival (OS) analysis of all 200 patients analyzed, comparing those patients having deletion 13 (*P* = 0.01) or translocation 4;14 (*P* = 0.0001) in their BM-lymphocytes with those lacking these abnormalities. The survival comparison also showed a trend toward reduced survival for patients having BA positive, trisomy1, and deletion of 14q32 (with 4;14 probe) (*P* = 0.08). Panel B: OS analysis comparing patients having the indicated abnormality only in their PC with patients having the same abnormality in both PC and lymphocytes (statistically significant only for deletion 13 (*P* = 0.002), trisomy 1 (*P* = 0.001), with a trend for t(4;14) (*P* = 0.08). Panel C: Aggregate OS analysis of 135 patients who had at least one abnormality in their PC, comparing patients having any abnormality present only in the PC with patients having any abnormality in both PC and lymphocytes (*P* = 0.01). Correlation between abnormal BM-lymphocytes and other clinical parameters: Univariate analysis showed that if deletion 13 was present in the lymphocytes, then the OR of having light chain (LC) present in urine versus not having light chain is 3.17, with 95% confidence interval (CI). Likewise, the OR of having higher levels of creatinine versus lower levels of creatinine is 5.07, 95% CI, if the patient harbors del13 in BM lymphocytes. In contrast, the presence of trisomy 17 in the lymphocytes inversely correlated with a higher amount of B2 microglobulin OR: 0.03, 95% CI. These correlations remained significant in the multivariate analysis. The presence of multiple abnormalities in PC and/or lymphocytes did not add any significance to the above correlations.

First, we compared all 200 patients, whether or not they had chromosomal abnormalities in their PCs, with each subset of patients who had at least one abnormality their BM-lymphocytes (each abnormality separately). Those patients having deletion 13 or t(4;14) in their BM-lymphocytes have a significantly shorter overall survival (OS) than patients without either of these abnormalities in BM-lymphocytes [*P* = 0.01, HR = 2.40 for del13; *P* = 0.0001, HR = 5.36 for t(4;14); Fig. 5, panel A].

We then compared the OS of patients that have abnormalities in their PCs but no detectably abnormal lymphocytes, with the survival of patients who have abnormal PC and also harbor chromosomally abnormal BM-lymphocytes (Fig. 4B) for each abnormality separately. For patients with the same abnormality in their PCs, only BM-lymphocytes harboring deletion 13 (*P* = 0.001, HR = 5.52) or trisomy 1 (*P* = 0.001, HR = 5.71; Fig. 5B), with a trend for t(4;14), confer a statistically significant reduced survival when compared with those patients having one or the other of these abnormalities *only* in PC.

The presence of multiple abnormalities in PC and/or BM-lymphocytes did not statistically influence survival.

Combining all patients with at least one chromosomal abnormality in their PCs, independent of treatment(s), we divided them into two groups: the group having abnormalities only in their PCs was compared with a second group having abnormalities in *both* PC and BM-lymphocytes. We find that patients with abnormalities present in both PC and BM-lymphocytes had a significantly worse OS than those patients with abnormalities present only in PC (*P* = 0.01, HR = 2.68; Fig. 5, panel C), regardless of the abnormality detected.

## Discussion

Using stringent morphologic criteria to define lymphocytes and PC, we show here that for all analyzed MM patients having at least one chromosomal abnormality in their PCs, 45% also have at least one such abnormality in their BM-lymphocytes, shown for a subset of patients to include CD20+ cells. This approach avoids any need to make assumptions about the phenotypes comprising the MM clone, given that purification of B-cells potentially excludes important sets of CSC. We screened BM-lymphocytes for IgH translocations, deletions/amplifications, and numerical abnormalities and identified a total of 17 different numerical or structural abnormalities in PC, nearly all of which were also detected in BM-lymphocytes of at least some patients. Furthermore, analysis of OS revealed that patients who had at least one chromosomal abnormality in both BM-lymphocytes and PC had significantly worse survival than did those patients having abnormalities only in their PCs. When all 200 patients were included in the analysis, regardless of the chromosomal status of their PCs, patients with at least one abnormality in their BM-lymphocytes still showed significantly worse survival independent of treatment type(s). Based on individual chromosomal abnormalities, only del13, translocation t(4;14), and trisomy1 showed a correlation with OS. The presence of chromosomal abnormalities in BM-lymphocytes provides strong evidence for their participation in the malignant process in MM, perhaps as CSC, as well as a marker for their identification. The presence or absence of any particular chromosomal abnormality may be of less clinical significance than the fact that abnormal lymphocytes populate the BM, and their presence correlates with reduced survival.

For the most part, BM-lymphocytes had only one abnormality, always reflecting the same abnormality in PC from the same slide, but for some patients as many as four distinct abnormalities were detected in their BM-lymphocytes, all shared with autologous PC. BM-lymphocytes comprise multiple subsets with specific shared morphologic characteristics, but only some (from 2 to 37%) are shown to be MM-related lymphocytes. Overall, numerical abnormalities, especially hyperdiploidy, were the most frequent among BM-lymphocytes (9–71%) in terms of the number of patients involved. IgH translocations were frequent (23–30% of patients) but usually comprised a smaller proportion of lymphocytes (2–20%) in any given patient. Del13 was frequent (25.5% of patients, in 6–30% of lymphocytes), but del17 (p53) and amplification 1q21 were never detected in BM-lymphocytes, despite the fact that those same lymphocytes do harbor numerical abnormalities in ch1 and ch17 for 31–33% of patients. Among lymphocytes, numerical abnormalities in ch1 and ch17 indicate that aberrant events, likely during mitosis, do affect ch1 and ch17, but are divorced from the deletion and amplification events that lead to del17(p53) and amp1q21. Our work suggests that these latter abnormalities, amp1q21 and del17(p53), are generated only in PC, consistent with their categorization by others as progression events during MM evolution [41–43]. In lymphocytes, among the abnormalities detected are those thought to be early stage biomarkers for MM (IgH translocations, hyperdiploidy, and del13), some of which are found in MGUS [1, 43, 44].

A BM aspirate includes white blood cells physically present at a defined geographical location in the body. Thus, BM-lymphocytes were in relatively close proximity to their presumed progeny, the BM-PC. Analysis of BM architecture shows localized clusters of lymphocytes adjacent to PC, suggesting a lineage relationship [44, 45], supported by 3D culture model within which self-renewing CD20+ clonotypic lymphocytes from BM ultimately gave rise to PC [44], as expected for putative CSC. Although we and others [35, 46, 47] have described clonotypic B cells in the circulation of MM patients, and chromosomal abnormalities have been detected in circulating MM B cells, there have been few reports characterizing BM-lymphocytes with respect to clonal characteristics shared with MM-PC. Our observation that lymphocytes and PCs in the same aspirate share chromosomal abnormalities provide evidence for a lineage relationship between these two compartments of the MM clone, suggesting that many chromosomal abnormalities may arise in lymphocytes and are passed to their PC progeny or the unlikely possibility that a subset of MM-PC reverts to lymphocyte morphology. This latter possibility seems excluded by the absence of deletion p53 and amp1q21 in BM-lymphocytes. The absence of del17(p53) and amp1q21 from BM-lymphocytes suggests that at some point, the PC compartment of the MM clone becomes self-sustaining, giving rise to clonal expansions harboring markers not found in BM-lymphocytes.

Of 135 patients with at least one abnormality in their PC, 45% (61) also had at least one abnormality in their BM-lymphocytes. This is almost certainly an underestimate of the frequency with which lymphocyte abnormalities are present, because the test only detects abnormal cells rising above a threshold or cut-off value for that probe set. The cutoff represents the number of “false positives” among healthy lymphocytes and is heavily dependent upon the probes used. Internal controls on each slide validated the analysis of BM-lymphocytes, with autologous PC providing the positive control and autologous M/PMNs providing negative control populations on the same slide and ensuring specificity of the testing. BM includes ∼11–24% lymphocytes, many *un*related to the MM clone. Moreover, if the number of clonotypic aberrant B cells is below the test threshold, they will remain undetectable, even though they may have *bona fide* chromosomal abnormalities. Our results are informative but likely to underestimate the true incidence of chromosomally abnormal BM-lymphocytes.

For a subset of 75 patients, we tested all probes to evaluate both PCs and BM-lymphocytes. In this set, 58% of patients with at least one abnormality also had the same abnormality(s) in their BM-lymphocytes. PCs had from only one to as many as eight distinct chromosomal abnormalities. Autologous lymphocytes had as many as four distinct abnormalities. The more abnormalities that are present in the PCs, the greater the likelihood of detecting at least one abnormality in the BM-lymphocytes. For patients with more than one chromosomal abnormality in their PCs, about half also had more than one abnormality in their BM-lymphocytes.

Some patients had PCs bearing four to seven signals for chromosome 11, but the maximum among their BM-lymphocytes was three signals for ch11 (except one case with four). This suggests that the extent of mitotic dysregulation required to gain four to seven copies of ch11 may occur only in PC and by extension only at later stages of disease. A variety of evidence suggests that PCs eventually acquire some degree of replicative autonomy that no longer involves lymphocytes. In this context, it is relevant that PCs express high levels of RHAMM, an oncogene that binds to the centrosome and mediates instability of the mitotic spindle [48] leading to lagging chromosomes and chromosomal missegregation [49]. RHAMM-mediated chromosomal abnormalities may eventually contribute to the outgrowth of chromosomally abnormal PCs that are likely to have only limited generative potential [39].

Given the compelling evidence that lymphocytes comprise a clinically important compartment of the MM clone and may include CSC, we suggest that in overt MM, clonal expansion may simultaneously occur on at least two levels, first that of the B cells, which persistently give rise to PC, and second that of PC themselves as they continue clonal expansion in the absence of contributions from the B cells. Abnormalities such as del13, hyperdiploidy, or IgH translocations may characterize initial clonal expansion of MM, maintained by clonotypic and chromosomally abnormal lymphocytes with extended generative potential [39], which may be MM CSC, while del17(p53) or amplification of 1q21 may occur as progression events restricted to PC. Furthermore, the presence of abnormalities in the lymphocyte compartment confers an unfavorable prognosis for affected patients, either because is reflective of a more aggressive disease and/or because this compartment represents a drug-resistant reservoir of CSC that maintains the disease. The presence of chromosomally abnormal BM-lymphocytes confirms their intimate clonal relationship with MM PC and supports the idea that MM lymphocytes are key contributors throughout malignant progression.
